# Mac-2 binding protein glycosylation isomer (M2BPGi) to evaluate liver fibrosis and cancer in HBV-infected patients in West Africa

**DOI:** 10.7189/jogh.12.04076

**Published:** 2022-11-12

**Authors:** Jeanne Perpétue Vincent, Gibril Ndow, Shintaro Ogawa, Amie Ceesay, Ramou Njie, Bakary Sanneh, Ignatius Baldeh, Umberto D’Alessandro, Maimuna Mendy, Mark Thursz, Isabelle Chemin, Yasuhito Tanaka, Maud Lemoine, Yusuke Shimakawa

**Affiliations:** 1 Unité d'Épidémiologie des Maladies Émergentes, Institut Pasteur, Paris, France; 2 Division of Digestive Diseases, Department of Metabolism, Digestion & Reproduction, Imperial College London, London, United Kingdom; 3 Disease Control & Elimination, MRC Unit The Gambia at London School of Hygiene & Tropical Medicine, Fajara, The Gambia; 4 Department of Virology and Liver Unit, Nagoya City University Graduate School of Medical Sciences, Nagoya, Japan; 5 Edward Francis Small Teaching Hospital, Banjul, The Gambia; 6 School of Medicine & Allied Health Sciences, University of The Gambia, Serekunda, The Gambia; 7 National Public Health Laboratories, Ministry of Health, Serekunda, The Gambia; 8 International Agency for Research on Cancer (IARC), World Health Organization, Lyon, France; 9 INSERM U1052, CNRS UMR5286, Centre de Recherche en Cancérologie, Université Claude Bernard, Lyon, France; 10 Department of Gastroenterology and Hepatology, Faculty of Life Sciences, Kumamoto University, Kumamoto, Japan; 11 International Research Center for Medical Sciences (IRCMS), Kumamoto University, Kumamoto, Japan

## Abstract

**Background:**

To reduce mortality associated with hepatitis B virus (HBV) infection, timely detection of cirrhosis and early-stage hepatocellular carcinoma (HCC) is essential. In low-income countries, however, HBV-infected people have limited access to liver histopathology, a reference test. Recently, Asian studies have suggested the usefulness of an inexpensive serum biomarker called Mac-2 binding protein glycosylation isomer (M2BPGi) in staging liver fibrosis and predicting HCC in HBV-infected patients.

**Methods:**

We systematically searched PubMed for studies examining the performance of M2BPGi in staging liver fibrosis in HBV-infected people, published up to September 21, 2021, to elucidate the knowledge gap. We then conducted a cross-sectional study of 339 HBV-infected patients in The Gambia (cirrhosis  = 65, HCC = 73, non-cirrhosis non-HCC = 201). We evaluated the association of M2BPGi with cirrhosis and HCC by computing odds ratios (ORs) derived from logistic regression. We also assessed the performance of M2BPGi to stage liver fibrosis in 49 patients who underwent liver biopsy (derivation set) and 217 patients with transient elastography (validation set). Using the derivation set we drew the receiver operating characteristics (ROC) curves to identify optimal M2BPGi thresholds to indicate significant fibrosis and cirrhosis using biopsy as a reference. We then applied these cut-offs to the validation set to obtain its sensitivity and specificity for indicating significant fibrosis and cirrhosis using transient elastography as a reference.

**Results:**

The systematic review identified 13 studies, all of which were conducted in East Asia and none in Africa. In The Gambia, positive M2BPGi was significantly associated with both cirrhosis (adjusted OR = 7.8, 95% CI = 3.1-19.7) and HCC (adjusted OR = 10.1, 2.6-40.2). The areas under the ROC curve (AUROC) in the derivation and validation set were 0.62 and 0.78, respectively, to diagnose significant fibrosis, and 0.80 and 0.89, respectively, to diagnose cirrhosis. By applying the optimal cut-offs, the sensitivity and specificity in the validation set were 61.5% and 93.4%, respectively, to diagnose significant fibrosis, and 72.5% and 92.2%, respectively, for cirrhosis.

**Conclusions:**

To the best of our knowledge, this is the first evaluation of M2BPGi in HBV-infected African population. The findings supported its accuracy in the diagnosis of cirrhosis in HBV-infected patients in West Africa.

Hepatitis B virus (HBV) infection is a significant global health problem. Worldwide, 296 million people are estimated to live with chronic HBV infection (CHB), of whom around 820 000 die yearly due to liver complications including cirrhosis and hepatocellular carcinoma (HCC) [1]. Over 20% of the global HBV burden is carried by sub-Saharan Africa (SSA) where an estimated 6.1% of the general population lives with CHB [2]. In 2019, an estimated 80 000 people died of CHB in SSA alone [1].

The World Health Organization (WHO) estimates that in Africa only 2% of people chronically infected with HBV are aware of their infection and only 0.1% are receiving antiviral treatment in 2019 [1]. This low uptake of HBV diagnosis affects not only the timely administration of antiviral therapy to prevent liver complications in patients with CHB, but also the timely detection of cirrhosis and early-stage hepatocellular carcinoma (HCC) [3,4]. In SSA, the majority of liver tumours present at a late stage, where curative treatment would be extremely challenging [5,6].

The gold standard test for staging liver fibrosis and diagnosing HCC is a liver histopathology [7,8]. However, the access to liver biopsy is severely limited in SSA because of high costs, discomfort to the patient, the risks of complications like bleeding, the possibility of sampling error, and the need of trained liver histopathologists and sophisticated infrastructure [9]. Thus, alternative non-invasive methods based on imaging or serum biomarkers are highly warranted [10]. For the diagnosis of significant fibrosis and cirrhosis, transient elastography (FibroScan®) has largely replaced liver biopsy particularly in high-income countries [7]. However, since transient elastography remains costly (US$ 34 000 for the portable machine and US$ 8500 for the yearly maintenance) [9], its access is severely limited for the majority of African patients [11,12]. WHO recommends the use of aspartate aminotransferase (AST)-to-platelet ratio index (APRI) to diagnose cirrhosis in low-income and middle-income countries (LMIC) but its diagnostic accuracy has been reported to be suboptimal [13]. Alternatively, the use of the gamma-glutamyl transpeptidase (GGT) to platelet ratio (GPR) has been suggested with a moderate accuracy for staging liver fibrosis [14,15]. Imaging-based diagnosis of HCC requires contrast-enhanced techniques using computed tomography (CT) scans or magnetic resonance imaging (MRI) [8], both of which are not widely available in SSA.

Mac-2 Binding Protein Glycosylation isomer (M2BPGi) is a novel serum glycoprotein-based marker for liver fibrosis progression [16]. Glycoproteomic studies found that progression of fibrosis leads to a specific modification of the glycosylation and sugar chain structure on the Mac-2 binding protein (M2BP). An assay to quantify the amount of M2BP with altered glycan structure has been developed in Japan [17] and approved as an in vitro diagnostic to evaluate the fibrosis stage since 2015 [18]. The assay has been found to have high accuracy to diagnose fibrosis stage among patients with chronic hepatitis C virus (HCV) infection; subsequently M2BPGi has been evaluated in other liver diseases with diverse aetiologies including CHB, non-alcoholic fatty liver disease, and primary biliary cirrhosis [19,20]. Its utility has been proven to indicate the presence of liver fibrosis [21,22], and to predict the development of HCC [23] and its recurrence [24] in patients with CHB. Through a fully automated immunoanalyzer, this marker can be measured in 17 minutes using 10 μL of serum, and the reagent costs only US$ 7/assay [17]. However, most of these studies were conducted in resource-rich Asian countries and it is uncertain whether this marker may be useful in CHB patients living outside of Asia.

In order to elucidate the knowledge gap, we systematically searched PubMed for studies examining the performance of M2BPGi in CHB patients. We then conducted a cross-sectional study and quantified M2BPGi in well-characterized CHB patients from The Gambia, West Africa, to answer the following questions. Is M2BPGi independently associated with cirrhosis or HCC in African patients with CHB? How well does M2BPGi perform in diagnosing significant liver fibrosis or cirrhosis in this population?

## METHODS

### Systematic review

We systematically searched PubMed for studies examining the sensitivity and specificity of M2BPGi to diagnose significant fibrosis or cirrhosis in CHB patients, published up to September 21, 2021. We used the following search terms and their variation: “hepatitis B” AND “M2BPGi” (Search strategy in the **Online Supplementary Document**). After the screening of titles and abstracts, we performed full-text reading and extracted the following data from the included studies: country, study design, reference test, characteristics of study participants (concurrent anti-HBV therapy, fibrosis stage), method to select M2BPGi cut-offs, and the diagnostic performance (area under the receiver operating characteristic curve (AUROC), sensitivity, specificity).

### Study participants in The Gambia

We used serum samples collected as part of the Prevention of Liver Fibrosis and Cancer in Africa (PROLIFICA) program in The Gambia for the current analysis of M2BPGi. Briefly, in 2011-2014, the program recruited a cohort of treatment-naïve adults found to carry hepatitis B surface antigen (HBsAg) through community-based and blood bank screening using a rapid test (Determine, Alere, or OnSite Combo Rapid Test, CTK Botech) [25,26]. The program also recruited a cohort of symptomatic patients with suspected chronic liver diseases referred from clinics across the country [27,28]. Following an informed consent, blood samples were obtained for biochemistry (VITROS 350 analyser, Ortho, USA), haematology (Medonic SE-12613, Boule Medical AB, Sweden), hepatitis B e antigen (HBeAg, ETI-EBK Plus, Diasorin, Italy), antibody against HCV (anti-HCV) (AxSYM, Abbott, USA), antibody against hepatitis D virus (ETI-AB-DELTAK-2, Diasorin, Italy), antibody against HIV (anti-HIV) (Genscreen ULTRA, Biorad, USA), and HBV DNA using a quantitative in-house PCR (detection limit 50 IU/mL) [29]. Hepatitis B core-related antigen (HBcrAg) was quantified using a chemiluminescent immunoassay (Lumipulse G600II, Fujirebio, Tokyo, Japan) [30]. In addition, fasting transient elastography (FibroScan® 402, Echosens, France) [31] and abdominal ultrasonography were systematically performed. Liver biopsy was conducted in a subset of the patients [14]. All participants have given a written consent before their inclusion. The current analysis included HBsAg-positive patients consecutively recruited from April 2012 to October 2013, and excluded those without liver stiffness measurement. The study was approved by The Gambian Government and Medical Research Council Joint Ethics Committee and reported in accordance with Standards for Reporting Diagnostic Accuracy (STARD) [32].

### Case definition

For the analysis of HCC as a clinical outcome, the HBV-infected patients were classified into two groups: i) HBsAg-positive people without HCC; and ii) HBsAg-positive HCC cases. The latter was defined histopathologically or clinically (a focal liver lesion ≥2 cm consistent with HCC by ultrasound and alpha-fetoprotein (AFP) levels ≥200 ng/mL) [28]. A previous case-control study of HCC in The Gambia rigorously evaluated the cut-off value of AFP to define HCC cases in the presence of a focal live lesion using ultrasound and determined the optimal threshold being ≥100 ng/mL using liver histopathology as a reference [33]. In order to assure high specificity, we used a higher cut-off value of AFP (≥200 ng/mL) to define HCC in the current analysis.

For the analysis of significant liver fibrosis and cirrhosis as clinical outcomes, we excluded HCC patients and divided them into two groups: those who had liver biopsy and those who had transient elastography without liver biopsy. The former group was used as a derivation data set to select the optimal cut-offs for the M2BPGi to maximize the sum of sensitivity and specificity to diagnose significant fibrosis (defined as Metavir score≥F2) or cirrhosis (defined as Metavir F4). To validate these cut-offs of M2BPGi obtained through the derivation data set, the latter group was used as a validation data set to estimate the sensitivity and specificity of M2BPGi. In the validation data set, significant fibrosis was defined as liver stiffness ≥7.9 kPa and cirrhosis as ≥9.5 kPa. These transient elastography thresholds were locally determined as being the most optimal during a previous cross-sectional evaluation of a cohort of HBV-infected individuals in The Gambia and used liver histopathology as a gold standard test [14].

### Serum M2BPGi

Sera were stored at -80°C and shipped to Toshiba General Hospital, Tokyo, Japan. M2BPGi was quantified using a sandwich immunoassay automated using the HISCL-800 system (Sysmex Co., Hyogo, Japan) according to the manufacturer’s instructions. The measurements were performed by laboratory staff unaware of the participants’ clinical status. The results analysed for M2BPGi were presented as a cut-off index (COI) calculated as follows:

M2BPGi COI = (M2BPGi_sample_ – M2BPGi_negative control_) / (M2BPGi_positive control_ – M2BPGi_negative control_)

The assay has a reportable range from 0.10 to 20.00 COI and a value of ≥1.00 COI was considered as positive [18,34].

### Statistical analyses

Continuous variables were summarized as a median (interquartile range (IQR)), and categorical variables were summarized as a frequency (percentage). Logistic regression was used to evaluate whether M2BPGi was independently associated with cirrhosis or HCC. Following variables were considered as a priori confounders: sex, age group, HBV DNA levels, viral genotypes, ALT, and platelet count. Those significantly associated with the liver diseases (*P*-value <0.05) in univariable analyses were further included in multivariable models. The discrimination capabilities of M2BPGi for significant liver fibrosis (Metavir F0-1 vs≥F2 in the derivation set, and liver stiffness measurement <7.9 vs ≥7.9 kPa in the validation set) and cirrhosis (Metavir F0-3 vs F4 in the derivation, and liver stiffness <9.5 vs ≥9.5 kPa in the validation) were analysed using the AUROC. The optimal cut-offs of M2BPGi obtained from the derivation set were applied to the validation set to estimate sensitivity and specificity. Stata 14.0 (StataCorp, TX, USA) was used for the analyses.

## RESULTS

### Systematic review

Of 51 articles identified, 13 met our inclusion criteria. **Table 1** presents the characteristics of these studies. All these studies were conducted in East Asia (5 in Japan, 3 in Taiwan, 2 in China, 2 in Korea and 1 in Hong Kong) and none in Africa. All evaluated the accuracy of M2BPGi through a cross-sectional design. As a reference standard, all used liver biopsy, except one [21] which used transient elastography. To diagnose significant fibrosis (≥F2), the AUROC of M2BPGi varied from 0.58 and 0.90 with a median value of 0.71 (**Table 1**). To diagnose cirrhosis (F4), the AUROC varied from 0.61 and 0.91 with a median value of 0.72. The majority of the studies applied an optimal cut-off of M2BPGi derived from each data set that maximized the sum of sensitivity and specificity (ie, Youden Index), and none but one [43] externally validated the cut-off obtained in an independent cohort. As a result, considerable variation was observed in the cut-off values used to evaluate its performance, ranging between 0.25 and 1.40 COI to diagnose significant fibrosis (≥F2), and between 0.70 and 2.00 COI to diagnose cirrhosis (**Table 1**).

**Table 1:** Previous studies evaluating the sensitivity and specificity of M2BPGi to diagnose significant fibrosis or cirrhosis in HBV-infected patients

Author	Year	Country	Gold standard	Anti-HBV therapy (%)	N	Fibrosis stage, n (%)	Cut-off selection method	To diagnose significant fibrosis (≥F2)				To diagnose cirrhosis (F4)			
**M2BPGi cut-off (COI)**	**AUC**	**Sen (%)**	**Spe (%)**	**M2BPGi cut-off (COI)**	**AUC**	**Sen (%)**	**Spe (%)**
**Wu** [35]	**2020**	Taiwan	LB (Metavir)	N/R	135	N/R	Youden index	1.11	0.58	60.3	64.1	1.07	0.61	65.3	55.8
**Tsuji** [36]	**2020**	Japan	LB (Metavir)	None	96	F0 = 25 (26.1), F1 = 44 (45.8), F2 = 14 (14.6), F3 = 10 (10.4), F4 = 3 (3.1)	N/R	0.89	0.90	92.6	82.4	N/R			
**Yeh** [22]	**2019**	Taiwan	LB (Metavir)	N/R	160	F0 = 21 (13.1), F1 = 51 (31.9), F2 = 37 (23.1), F3 = 25 (15.6), F4 = 26 (16.3)	Youden index	1.35	N/R	65.9	80.6	1.67	N/R	69.2	76.1
**Chen** [37]	**2019**	Taiwan	LB (Metavir)	N/R	33	N/R	N/R	N/R	0.58	85.2	50.0	N/R	0.69	84.6	65.0
**Mak** [38]	**2018**	Hong Kong	LB (Ishak)	41%*	327†	F0-1 = 292 (52.7), F2 = 206 (37.2), F3 = 50 (9.0), F4 = 6 (1.1)	Youden index	0.25	0.65	74.8	47.3	0.96	0.91	83.3	92.7
**Wei** [21]	**2018**	China	Fibro Scan	N/R	228	F0-1 = 127 (55.7), F2-3 = 32 (14.0), F4 = 69 (30.3)	N/R	1.12	0.79	72.3	73.2	1.83	0.81	55.0	93.7
**Ishii** [39]	**2017**	Japan	LB (Metavir)	None	189	F0 = 11 (5.8), F1 = 97 (51.3), F2 = 37 (19.6), F3 = 28 (14.8), F4 = 16 (8.5)	Youden index	1.40	0.77	67.9	74.1	1.90	0.87	87.5	80.4
**Jekarl** [40]	**2017**	Korea	LB (Knodell)	None	151	F0 = 8 (5.3), F1 = 86 (57.0), F3 = 42 (27.8), F4 = 15 (9.9)	Youden index	0.70	0.66	50.8	70.7	0.70	0.72	73.3	62.5
**Noguchi** [41]	**2017**	Japan	LB (Metavir)	N/R	70	F0 = 9 (12.8), F1 = 25 (35.7), F2 = 17 (24.3), F3 = 13 (18.6), F4 = 6 (8.6)	N/R	0.81	N/R	50.0	48.9	N/R			
**Ichikawa** [42]	**2016**	Japan	LB (Metavir)	None	112	F0 = 4 (3.6), F1 = 36 (32.2), F2 = 26 (23.2), F3 = 24 (21.4), F4 = 22 (19.6)	Youden index	0.94	0.71	68.0	72.0	1.26	0.68	68.0	69.0
**Zou** [43] (training)	**2016**	China	LB (Metavir)	None	221	F0 = 35 (15.8), F1 = 97 (43.9), F2 = 42 (19.0), F3 = 23 (10.4), F4 = 24 (10.9)	Youden index	1.06	0.75	60.5	79.8	N/R			
**Zou** [43] (validation)					76	F0 = 10 (13.2), F1 = 29 (38.1), F2 = 17 (22.3), F3 = 10 (13.2), F4 = 10 (13.2)	From the training set	1.06	N/R	59.5	82.1				
**Nishikawa** [44]	**2016**	Japan	LB (Metavir)	24%	249	F0 = 14 (5.6), F1 = 124 (49.8), F2 = 51 (20.5), F3 = 41 (16.5), F4 = 19 (7.6)	Youden index	1.37	0.73	60.4	74.6	1.86	0.78	73.7	80.0
**Heo** [45]	**2016**	Korea	LB (Battsand Ludwig)	82%	95	F0-1 = 16 (16.8), F2 = 29 (30.5), F3 = 10 (10.6), F4 = 40 (42.1)	Youden index	0.80	0.69	87.3	43.8	2.00	0.70	35.0	92.7

AUC – area under the receiver operating characteristics curve, COI – cut-off index, LB – liver biopsy, M2BPGi – Mac-2 binding protein glycosylation isomer, N/R – not reported, Sen – sensitivity, Spe – specificity

*Percentage of samples obtained during antiviral therapy.

†554 samples from 327 patients were analysed.

### Characteristics of the study participants in The Gambia

**Figure 1:**
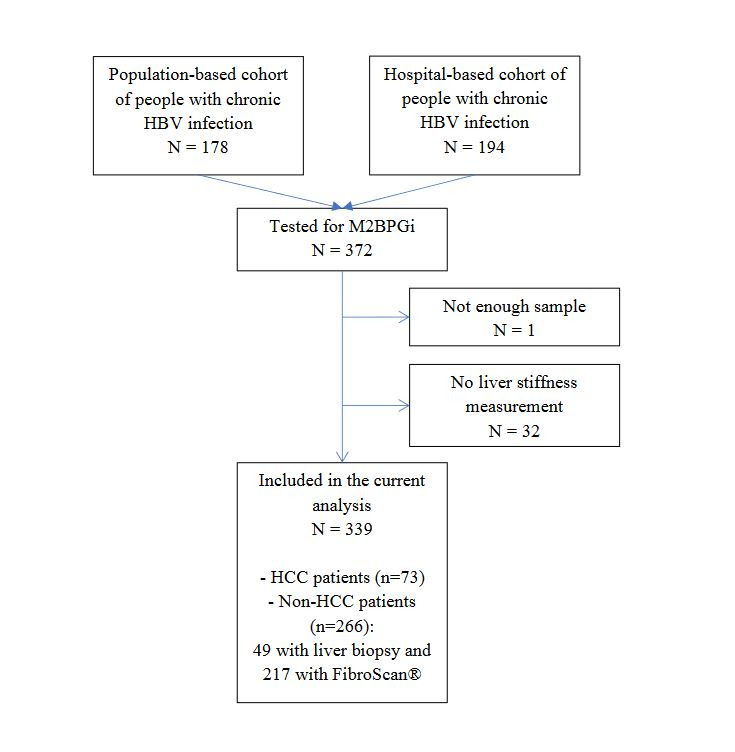
Flowchart of study participants.

A flow diagram of study participants is presented in **Figure 1**. A total of 339 HBsAg-positive patients were included in this cross-sectional study; 73 had HCC at the time of diagnosis and 266 did not have HCC. In the latter group, 49 participants underwent liver biopsy (ie, the derivation set) and 217 had transient elastography without biopsy (ie, the validation set). Their characteristics are presented in **Table 2**. The median age of the study participants was 38 (IQR = 31-48) years and 67.6% (229/339) were men. Those with HCC were older and more frequently males than those without HCC (**Table 2**). There was a significant difference between the groups for the values of biochemistry and haematology markers. Prevalence of positive M2BPGi (≥1.00 COI) was significantly higher in the HCC group (94.5%, 69/73) than in those without HCC (48.5%, 129/266, *P* < 0.001). The median value of M2BPGi was significantly higher in HCC (COI = 2.56, IQR = 1.63-4.75) than in non-HCC group (COI = 0.97, IQR = 0.53-1.75) (*P* < 0.001).

**Table 2:** Characteristics of the study participants by the disease status (n = 339)

Variables	All (n = 339)	HBsAg-positive patients without HCC		HBsAg-positive patients with HCC (n = 73)	*P-*value*
**With liver biopsy (derivation set) (n = 49)**	**With transient elastography (validation set)** **(n = 217)**
**Demographic variables**					
Median age (IQR), years	38 (31-48)	33 (27-38)	38 (31-49)	41 (32-52)	<0.001
Male sex, n (%)	229 (67.6)	43 (87.8)	131 (60.4)	55 (75.3)	<0.001
Ever drunk alcohol, n (%)	27 (8.0)	3 (6.1)	21 (9.8)	3 (4.2)	0.16
Family history of liver cancer, n (%)	13 (3.8)	4 (8.2)	8 (3.7)	1 (1.4)	0.40
**Liver disease markers**					
Median AST (IQR), IU/L	35 (27-112)	41 (31.0-55.5)	30 (25.0-43.5)	216 (163-427)	<0.001
Median ALT (IQR), IU/L	28 (21-50)	36 (25-46)	24 (18-34)	61 (37-113)	<0.001
Median GGT (IQR), IU/L	36 (22-169)	42.5 (27.5-70.5)	28 (20-47)	354 (219-594)	<0.001
Median albumin (IQR), g/L	40 (33-43)	42 (40-45)	41 (36-43)	32 (28-37)	<0.001
Medial total bilirubin (IQR), IU/L	13 (9-22)	13 (8-16)	10.5 (8-15)	28.5 (18.5-93.0)	<0.001
Median platelet count (IQR), 10^9^/L	191 (138-256)	159 (131-207)	185 (134-248)	243.5 (173-346)	<0.001
Median M2BPGi (IQR), COI	1.17 (0.59-2.64)	0.85 (0.49-1.84)	0.98 (0.54-1.69)	2.56 (1.63-4.75)	<0.001
Median liver stiffness (IQR), kPa	7.6 (4.7-30.7)	4.9 (4.2-7.1)	27.7 (19.3-75.0)	75 (49.6-75.0)	<0.001
Alpha-fetoprotein (IQR), ng/mL	13.2 (5.5-742)	8.3 (5.0-12.7)	8.1 (3.7-19.0)	2942 (479-7364)	<0.001
**HBV markers**					
HBeAg positive, n (%)	51 (16.0)	8 (16.3)	21 (10.3)	22 (33.3)	<0.001
HBcrAg positive, n (%)	189 (55.8)	34 (69.4)	108 (49.8)	47 (64.4)	0.01
Median HBsAg levels (IQR), log_10_ IU/mL	3.5 (2.7-4.1)	3.7 (3.5-4.0)	3.5 (2.8-4.1)	3.2 (1.2-3.6)	<0.001
HBV DNA levels, n (%)					<0.001
Undetectable (<50 IU/mL)	129 (38.6)	12 (24.5)	104 (49.1)	13 (17.8)	
50-1 999 IU/mL	112 (33.5)	15 (30.6)	75 (35.4)	22 (30.1)	
2000199 999 IU/mL	43 (12.9)	12 (24.5)	15 (7.1)	16 (21.9)	
≥200 000 IU/mL	50 (15.0)	10 (20.4)	18 (8.5)	22 (30.1)	
HBV genotypes					0.001
A	52 (19.0)	5 (10.9)	27 (15.6)	20 (36.4)	
E	222 (81.0)	41 (89.1)	146 (84.4)	35 (63.6)	
**Co-infection**					
HIV positive, n (%)	10 (3.0)	0	4 (1.8)	6 (8.2)	0.009
HCV positive, n (%)	13 (3.9)	0	9 (4.2)	4 (5.7)	0.27
HDV positive, n (%)	15 (5.0)	2 (4.2)	8 (4.1)	5 (8.5)	0.38

ALT – alanine transaminase, AST – aspartate transaminase, HBcrAg – hepatitis B core-related antigen, HBeAg – hepatitis B e antigen, HBV – hepatitis B virus, HCV – hepatitis C virus, HDV – hepatitis D virus, HIV – human immunodeficiency virus, IQR – interquartile range, M2BPGi – Mac-2 binding protein glycosylation isomer, qHBsAg – quantitative hepatitis B surface antigen

*P-values were obtained using Kruskal-Wallis test for continuous variables and Pearson χ^2^ test for categorical variables.

Distribution of hepatitis B viral markers also differed between the HCC group and non-HCC group. Prevalence of positive HBeAg was significantly higher in the HCC group (33.3%, 22/66) than in those without (11.5%, 29/253) (*P* < 0.001). High HBV viral loads were more frequently observed in the HCC group: the proportion with ≥200 000 IU/mL, 2000-199 999 IU/mL, 50-1 999 IU/mL, and undetectable (<50 IU/mL) was 30.1%, 21.9%, 30.1%, and 17.8% in the HCC group and 10.7%, 10.3%, 34.5%, and 44.4% in non-HCC group, respectively (*P* < 0.001). HBcrAg was positive (≥3.0 log U/mL) in 64.4% (47/73) of HCC cases and 53.4% (142/266) of non-HCC group (*P* = 0.09). The prevalence of HBV genotype A was significantly higher in HCC cases (36.4%, 20/55) than in non-HCC group (14.6%, 32/219, *P* < 0.001).

### Association between M2BPGi and HCC

**Table 3** presents the odds ratios for the association between positive M2BPGi and HCC. In the crude analysis, M2BPGi was significantly associated with HCC (crude odds ratio (OR) = 30.9, 95% confidence interval (CI) = 10.8-88.2, *P* < 0.001). After adjusting for other variables found to be associated with HCC in the crude analysis (age group, HBV DNA levels, HBV genotype, ALT and platelet count), positive M2BPGi remained significantly associated with HCC (adjusted OR = 10.1, 95% CI = 2.6-40.2, *P* = 0.001).

**Table 3:** Association between M2BPGi and HCC in HBsAg-positive patients (n = 339)

Variables		HBsAg-positive patients without HCC (n = 266)	HBsAg-positive HCC patients (n = 73)	Crude analysis		Adjusted analysis*	
**OR (95% CI)**	***P*-value**	**OR (95% CI)**	***P*-value**
Sex	Men	174 (65.4%)	55 (75.3%)	1.0	0.111	N/A	N/A
	Women	92 (34.6%)	18 (24.7%)	0.6 (0.3-1.1)			
Age group	<40 years	166 (62.6%)	33 (45.2%)	1.0	0.008	1.0	0.172
	≥40 years	99 (37.4%)	40 (54.8%)	2.0 (1.2-3.4)		1.9 (0.8-4.9)	
HBV DNA (IU/mL)	<2000	206 (78.9%)	35 (48.0%)	1.0	<0.001	1.0	<0.001
	2000-199 999	27 (10.3%)	16 (21.9%)	3.5 (1.7-7.1)		6.6 (2.2-20.1)	
	≥200 000	28 (10.7%)	22 (30.1%)	4.6 (2.4-9.0)		6.7 (2.2-20.9)	
HBV genotype	E	187 (85.4%)	35 (63.6%)	1.0	<0.001	1.0	0.001
	A	32 (14.6%)	20 (36.4%)	3.3 (1.7-6.5)		5.3 (2.0-14.3)	
ALT (IU/L)	<40	192 (74.4%)	17 (25.4%)	1.0	<0.001	1.0	<0.001
	≥40	66 (25.6%)	50 (74.6%)	8.6 (4.6-15.9)		6.2 (2.4-16.0)	
Platelet count (10^9^/L)	<150	92 (34.6%)	9 (12.3%)	1.0	<0.001	1.0	<0.001
	≥150	174 (65.4%)	64 (87.7%)	3.8 (1.8-7.9)		20.5 (6.0-69.9)	
M2BPGi	Negative	137 (51.5%)	4 (5.5%)	1.0	<0.001	1.0	0.001
	Positive	129 (48.5%)	69 (94.5%)	30.9 (10.8-88.2)		10.1 (2.6-40.2)	

ALT – alanine transaminase, M2BPGi – Mac-2 binding protein glycosylation isomer, N/A – not applicable, OR – odds ratio, CI – confidence interval

*All the variables significantly associated with HCC (*P*-value <0.05) in the crude analyses were mutually adjusted in the multivariable model.

### Association between M2BPGi and liver fibrosis

**Figure 2:**
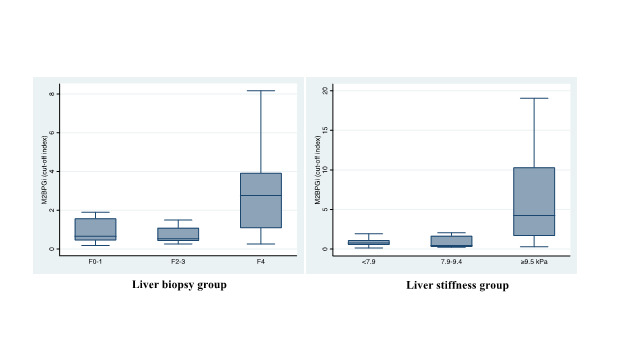
Distribution of M2BPGi levels by stage of fibrosis in patients with liver biopsy (n = 49, Metavir F0-1, F2-3, and F4) and in patients with transient elastography (n = 217, liver stiffness measurement <7.9, 7.9-9.4, and ≥9.5 kPa).

In 49 patients without the diagnosis of HCC who had liver biopsy, the number with Metavir score F0-1, F2-3, and F4 was 26 (53.0%), 9 (18.4%), and 14 (28.6%), respectively. In 217 patients without HCC who had transient elastography, liver stiffness was <7.9 kPa, 7.9-9.4 kPa, and ≥9.5 kPa in 152 (70.0%), 14 (6.5%), and 51 (23.5%), respectively. The distributions of M2BPGi levels, by the fibrosis stage in the derivation set and by the liver stiffness measurement in the validation set, are presented in **Figure 2**. In the liver biopsy group, the median of M2BPGi levels (COI) was 0.66 (IQR = 0.45-1.57), 0.53 (0.42-1.09), and 2.76 (1.08-3.92), in those with Metavir score of F0-1, F2-3, and F4, respectively (*P* = 0.0028). In the transient elastography group, the median value of M2BPGi (COI) was 0.77 (IQR = 0.52-1.11), 0.45 (0.27-1.67), and 4.25 (1.67-10.3), in those with liver stiffness measurement of <7.9 kPa, 7.9-9.4 kPa, and ≥9.5 kPa, respectively (*P* < 0.001).

To assess whether M2BPGi levels were associated with cirrhosis, the liver biopsy group and transient elastography group were combined (n = 266). The prevalence of positive M2BPGi was 87.7% (57/65) in patients with cirrhosis and 35.8% (72/201) in non-cirrhotic patients. In the crude analysis, positive M2BPGi was significantly associated with cirrhosis (OR = 12.8, 95% CI = 5.8-28.2, *P* < 0.001) (**Table 4**). After adjusting for age group, HBV DNA levels, ALT, and platelet count, positive M2BPGi was significantly associated with cirrhosis (adjusted OR = 7.8, 95% CI = 3.1-19.7, *P* < 0.001). Significant association was also observed between positive M2BPGi and significant fibrosis (Metavir score≥F2 or liver stiffness measurement ≥7.9 kPa) in the adjusted analysis, largely due to the inclusion of cirrhosis in this group (Table S1 in the **Online Supplementary Document**).

**Table 4:** Association between M2BPGi and cirrhosis in HBsAg-positive patients without HCC (n = 266)

Variables		HBsAg-positive patients without cirrhosis (n = 201)	HBsAg-positive patients with cirrhosis (n = 65)	Crude analysis		Adjusted analysis*	
**OR (95% CI)**	***P*-value**	**OR (95% CI)**	***P*-value**
Sex	Men	125 (62.2%)	49 (75.4%)	1.0	0.054	N/A	N/A
	Women	76 (37.8%)	16 (24.6%)	0.5 (0.3-1.0)			
Age group	<40 y	136 (67.7%)	30 (46.9%)	1.0	0.003	1.0	0.073
	≥40 y	65 (32.3%)	34 (53.1%)	2.4 (1.3-4.2)		2.1 (0.9-4.5)	
HBV DNA (IU/mL)	<2 000	167 (85.2%)	39 (60.0%)	1.0	<0.001	1.0	0.234
	2000-199 999	18 (9.2%)	9 (13.8%)	2.1 (0.9-5.1)		0.7 (0.2- 2.5)	
	≥200 000	11 (5.6%)	17 (26.2%)	6.6 (2.9-15.2)		2.4 (0.7-7.6)	
HBV genotype	E	146 (85.9%)	41 (83.7%)	1.0	0.700	N/A	N/A
	A	24 (14.1%)	8 (16.3%)	1.2 (0.5-2.8)			
ALT (IU/L)	<40	170 (85.9%)	22 (36.7%)	1.0	<0.001	1.0	<0.001
	≥40	28 (14.1%)	38 (63.3%)	10.5 (5.4-20.3)		5.6 (2.5-12.7)	
Platelet count (10^9^/L)	<150	55 (27.4%)	37 (56.9%)	1.0	<0.001	1.0	0.017
	≥150	146 (72.6%)	28 (43.1%)	0.3 (0.2-0.5)		0.4 (0.2-0.8)	
M2BPGi	Negative	129 (64.2%)	8 (12.3%)	1.0	<0.001	1.0	<0.001
	Positive	72 (35.8%)	57 (87.7%)	12.8 (5.8-28.2)		7.8 (3.1-19.7)	

ALT – alanine transaminase, M2BPGi – Mac-2 binding protein glycosylation isomer, N/A – not applicable, OR – odds ratio, CI – confidence interval

*All the variables significantly associated with cirrhosis (*P*-value <0.05) in the crude analyses were mutually adjusted in the multivariable model.

### Performance of M2BPGi for staging liver fibrosis

The performance of M2BPGi to diagnose significant fibrosis (≥F2 in the derivation set and ≥7.9 kPa in the validation set) and cirrhosis (F4 in the derivation set and ≥9.5 kPa in the validation set) are summarised in **Table 5**. The AUROC for M2BPGi to discriminate significant fibrosis was 0.62 (95% CI = 0.46-0.79) in the derivation set and 0.78 (0.70-87) in the validation set. The AUROC to discriminate cirrhosis was 0.80 (95% CI = 0.64-0.97) in the derivation and 0.89 (0.83-0.95) in the validation set. Using the optimal cut-off of M2BPGi derived from the derivation set (≥1.84 COI), its sensitivity and specificity to diagnose significant fibrosis were 61.5% (95% CI = 48.6-73.3) and 93.4% (95% CI = 88.2-96.8) in the validation set, respectively. The sensitivity and specificity to diagnose cirrhosis were 72.5% (95% CI = 58.3-84.1) and 92.2% (95% CI = 87.0-95.8) in the validation set, respectively.

**Table 5:** Performance of M2BPGi to diagnose significant fibrosis (≥F2 or ≥7.9 kPa) and cirrhosis (F4 or ≥9.5 kPa) in the derivation set (n = 49) and the validation set (n = 217)

	AUROC (95% CI)	Cut-off (COI)	Sensitivity (95% CI)	Specificity (95% CI)	PPV (95% CI)	NPV (95% CI)
**To diagnose significant fibrosis**						
**Derivation set (Metavir≥F2)**	0.62 (0.46-0.79)	1.84	43.5% (23.2-65.5)	88.5% (69.8-97.6)	76.9% (46.2-95.0)	63.9% (46.2-79.2)
**Validation set (≥7.9 kPa)**	0.78 (0.70-0.87)	1.84	61.5% (48.6-73.3)	93.4% (88.2-96.8)	80.0% (66.3-90.0)	85.0% (78.7-90.1)
**To diagnose cirrhosis**						
**Derivation set (Metavir F4)**	0.80 (0.64-0.97)	1.84	71.4% (41.9-91.6)	91.4% (76.9-98.2)	76.9% (46.2-95.0)	88.9% (73.9-96.9)
**Validation set (≥9.5 kPa)**	0.89 (0.83-0.95)	1.84	72.5% (58.3-84.1)	92.2% (87.0-95.8)	74.0% (59.7-85.4)	91.6% (86.3-95.3)

AUROC – area under the receiver operating characteristics curve, COI – cut-off index, M2BPGi – Mac-2 binding protein glycosylation isomer, NPV – negative predictive value, PPV – positive predictive value

## DISCUSSION

Our systematic review confirmed that all the previous studies that evaluated the performance of M2BPGi in people with CHB were conducted in East Asia and none in Africa. Our cross-sectional study of HBV-infected people in The Gambia, West Africa, found that M2BPGi was an independent factor associated with both cirrhosis (adjusted OR = 7.8, 95% CI = 3.1-19.7) and HCC (adjusted OR = 10.1, 95% CI = 2.6-40.2). In those without HCC, the AUROC for M2BPGi to discriminate significant fibrosis was modest with 0.62 (95% CI = 0.46-0.79) in the derivation set and 0.78 (95% CI = 0.70-87) in the validation set. However, the AUROC to discriminate cirrhosis was good with 0.80 (95% CI = 0.64-0.97) in the derivation and 0.89 (95% CI = 0.83-0.95) in the validation set. Using the optimal cut-off obtained from the derivation data set (≥1.84 COI), the sensitivity and specificity of M2BPGi in the validation set were 61.5% and 93.4%, respectively, to diagnose significant liver fibrosis, and 72.5% and 92.2%, respectively, to diagnose cirrhosis.

The role of M2BPGi to predict the development of HBV-related HCC has been extensively studied in Asia. In a longitudinal cohort study of 112 treatment-naïve CHB patients in Japan, Ichikawa et al. identified higher serum M2BPGi (≥0.71 COI) as an independent predictor for the development of HCC after a mean follow-up of 40 months (hazard ratio (HR): 8.3, 95% CI = 1.0-67.0) [42]. In Korea, Heo et al. found that the risk of HCC development in a longitudinal cohort of 95 CHB patients was 11.5 times higher (95% CI = 1.4-97.2) when they had elevated M2BPGi (≥1.80 COI) at baseline [45]. Using the same cut-off of M2BPGi (≥1.80 COI), another longitudinal study of 1323 CHB patients in Korea found that those with elevated M2BPGi at baseline (≥1.80 COI) had a higher risk of developing HCC (HR = 1.4, 95% CI = 1.1-1.8) after the median follow-up period of 60 months [23]. Of note, a subgroup analysis stratified by the presence of cirrhosis at baseline confirmed the higher rate of HCC incidence in those with elevated M2BPGi, irrespective of whether patients had cirrhosis. In United States and Taiwan, a longitudinal study of 714 CHB patients of Asian origin found that M2BPGi was a significant predictor of HCC development (HR of 1.11 in each increase in the unit of COI, 95% CI = 1.05-1.18) [46]. The association was also observed for the recurrence of HCC. Kim et al. evaluated the performance of M2BPGi to predict the HCC recurrence among CHB patients who underwent curative resection; they found that those having M2BPGi levels of >2.14 COI had higher risk of HCC recurrence [24]. Unlike those longitudinal studies in Asia which consistently supported its role in predicting the development of HCC, ours was a cross-sectional design, hampering the valid interpretation of the temporal sequence of the association between positive M2BPGi and HCC. In addition, most of our HCC cases presented with a highly advanced stage of the liver tumours and we could not establish whether the HCC have developed on a background of advanced fibrosis or not. Consequently, the association between M2BPGi and HCC could not be adjusted by the pre-existing cirrhosis, an important confounding factor, and we do not know whether the observed association merely reflected the advanced fibrosis stage in our HCC cases or there was an independent pathway between M2BPGi and HCC.

The association of M2BPGi with advanced fibrosis or cirrhosis has been also consistently shown in Asian patients with CHB. Unlike the studies evaluating HCC as an endpoint, most of these studies used a cross-sectional design. In Hong Kong, Mak et al. identified M2BPGi as the strongest independent variable that was significantly associated with the advanced fibrosis and cirrhosis (Ishak≥F3) in CHB patients (OR = 7.23, 95% CI = 2.32-22.48) [38]. In Japan, Ishii et al. found that the risk of significant fibrosis (≥F2) was 2.5 times (95% CI = 1.1-6.0) higher in CHB patients with M2BPGi ≥1.4 COI and the risk of cirrhosis (F4) was 6.4 times (1.0-95.1) higher in those with M2BPGi ≥1.9 COI [39]. Similar to these Asian studies, we observed in our African cohort that positive M2BPGi was significantly associated with≥F2 (adjusted OR = 4.0, 95% CI = 2.0-7.9) and F4 (adjusted OR = 7.8, 95% CI = 3.1-19.7).

In addition to its positive association, we found that the performance of M2BPGi to diagnose cirrhosis was high in CHB patients in The Gambia (AUROC of 0.80 in the derivation and 0.89 in the validation set) although its accuracy to diagnose significant fibrosis was suboptimal (AUROC of 0.62 in the derivation and 0.78 in the validation set), like most of non-invasive markers. This finding was in line with the AUROCs reported by the previous Asian studies identified through the systematic review (**Table 1**). In our cohort, the most optimal threshold was 1.84 COI to diagnose both≥F2 and F4. As our systematic review highlighted, the lack of a standard threshold and the use of different cut-offs to estimate sensitivity and specificity in these studies makes interpretation of the results extremely difficult. Additional studies to validate its use, in particular by applying the same standard cut-off value, are highly warranted for this marker to become useful in actual clinical practice.

Our study has some limitations. First, M2BPGi was measured in a laboratory in Japan using stored sera because the immunoanalyzer is not yet available in The Gambia. It would have been more informative to perform the assay in a local laboratory in The Gambia. Second, this was a cross-sectional evaluation; a prospective cohort study is warranted to better elucidate the role of M2BPGi to predict the development of HCC in CHB patients in Africa. Third, the sample size in the current analysis was limited; further analysis with a larger sample size is needed. Finally, we were unable to address potentially important confounders for the association between M2BPGi and advanced liver diseases, such as alcohol consumption, smoking, obesity and exposure to aflatoxin.

## CONCLUSIONS

Using the well-characterized samples from The Gambia, this study found that positive M2BPGi (≥1.00 COI) was associated with cirrhosis and HCC in CHB patients in sub-Saharan Africa. The study also suggested that M2BPGi might be useful tool to diagnose HBV-related cirrhosis in Africa population. In addition to the low reagent cost (US$ 7/assay), the operational characteristics of M2BPGi, such as the use of a fully automated immunoanalyzer, rapid turn round time (17 minutes) and a small quantity of sample required (10 μL), all favour its potential use in resource-limited context [17]. The application of technique to the use of dried blood samples (DBS) or lateral-flow rapid diagnostic test may further improve the access to the test. Further research is warranted for this tool to be able to contribute to the global hepatitis elimination goal.

## Additional material


Online Suplementary Document
