# Supplementary material for: Mac-2 binding protein glycosylation isomer (M2BPGi) to evaluate liver fibrosis and cancer in HBV-infected patients in West Africa
Source: J Glob Health. 2022 Nov 12;12:04076. doi: 10.7189/jogh.12.04076 (PMC9653177; doi:10.7189/jogh.12.04076)
Supplement: Online Suplementary Document [file jogh-12-04076-s001.pdf]

ONLINE SUPPLEMENTARY DOCUMENT

Title: Mac-2 Binding Protein Glycosylation isomer (M2BPGi) to evaluate liver fibrosis and cancer in HBV-infected patients in West Africa

Authors: Jeanne Perpétue Vincent, Gibril Ndow, Shintaro Ogawa, Amie Ceesay, Ramou Njie, Bakary Sanneh, Ignatius Baldeh, Umberto D'Alessandro, Maimuna Mendy, Mark Thursz, Isabelle Chemin, Yasuhito Tanaka, Maud Lemoine, Yusuke Shimakawa

Search strategy for a systematic review of studies assessing the sensitivity and specificity of M2BPGi to diagnose significant fibrosis or cirrhosis in patients with chronic HBV infection

We searched PubMed for articles published by September 21st 2021, using a combination of MeSH and text terms as follows:

1. "hepatitis b"[MeSH] OR "hepatitis b virus"[MeSH] OR hepatitis b[Text] OR type b hepatitis[Text] OR hepatitis type b[Text] OR hbv[Text] OR hep b[Text]

AND

2. Wisteria floribunda agglutinin positive mac-2 binding protein[Text] OR mac-2 binding protein glycosylation isomer[Text] OR WFA M2BP[Text] OR M2BPGi[Text]

We included studies that reported sensitivity and specificity of M2BPG1 to diagnose significant fibrosis or cirrhosis in HBV-infected population.

Supplementary Table 1. Association between M2BPGi and significant fibrosis in HBsAg-positive patients without HCC (N=266)

**Variables**		**HBsAg-positive patients without significant fibrosis (n=178)**	**HBsAg-positive patients with significant fibrosis (n=88)**	**Crude analysis**		**Adjusted analysis***	
				**OR (95% CI)**	**p-value**	**OR (95% CI)**	**p-value**
Sex	Men	103 (57.9)	71 (80.7)	1.0	<0.001	1.0	0.003
	Women	75 (42.1)	17 (19.3)	0.3 (0.2-0.6)		0.3 (0.1-0.7)	
Age group	<40 years	116 (65.2)	50 (57.5)	1.0	0.224	N/A	N/A
	≥40 years	62 (34.8)	37 (42.5)	1.4 (0.8-2.3)			
HBV DNA (IU/mL)	<2 000	151 (87.3)	55 (62.5)	1.0	<0.001	1.0	0.124
	2 000–199 999	15 (8.7)	12 (13.6)	2.2 (1.0-5.0)		0.8 (0.3- 2.3)	
	≥200,000	7 (4.0)	21 (23.9)	8.2 (3.3-20.4)		2.8 (1.0-8.2)	
HBV genotype	E	130 (86.1)	57 (83.8)	1.0	0.660	N/A	N/A
	A	21 (13.9)	11 (16.2)	1.2 (0.5-2.6)			
ALT (IU/L)	<40	154 (88.0)	38 (45.8)	1.0	<0.001	1.0	<0.001
	≥40	21 (12.0)	45 (54.2)	8.7 (4.6-16.3)		4.2 (2.1-8.8)	
Platelet count (10^9/L)	<150	46 (25.8)	46 (52.3)	1.0	<0.001	1.0	0.106
	≥150	132 (74.2)	42 (47.7)	0.3 (0.2-0.5)		0.6 (0.3-1.1)	
M2BPGi	Negative	113 (63.5)	24 (27.3)	1.0	<0.001	1.0	<0.001
	Positive	65 (36.5)	64 (72.7)	4.6 (2.6-8.1)		4.0 (2.0-7.9)	

Abbreviations: ALT, alanine transaminase; M2BPGi, Mac-2 Binding Protein Glycosylation isomer; N/A, not applicable; OR, odds ratio.

* All the variables significantly associated with significant liver fibrosis (*P* value <0.05) in the crude analyses were mutually adjusted in the multivariable model.
